# Impact of physical incompatibility on drug mass flow rates: example of furosemide-midazolam incompatibility

**DOI:** 10.1186/2110-5820-2-28

**Published:** 2012-07-13

**Authors:** Aurélie Foinard, Bertrand Décaudin, Christine Barthélémy, Bertrand Debaene, Pascal Odou

**Affiliations:** 1 Laboratoire de Biopharmacie, Pharmacie Galénique et Hospitalière, EA 4481, IFR114, Université Lille Nord de France, 3 rue du Professeur Laguesse, BP83, 59006, Lille, France; 2 Pharmacie, CHRU Lille, rue Philippe Marache, 59006, Lille, France; 3 Département d’Anesthésie et de Réanimation Chirurgicale, Inserm U1070, CHU Poitiers, 2 rue de la Milétrie, BP577, 86021, Poitiers, France

**Keywords:** Intravenous drug, Drug incompatibility, Visible particle, Mass flow rate, Drug loss

## Abstract

**Background:**

Patients in intensive care units receive many drugs simultaneously but through limited venous accesses. Several intravenous therapies have to be administered through the same catheter, thus increasing the risk of physicochemical incompatibility. The purpose of this work was to assess and to quantify the impact of physical incompatibility on the mass flow rates of drugs infused simultaneously to the patient, through an in vitro study.

**Methods:**

Furosemide-midazolam incompatibility was used to assess the impact of physical incompatibility on drug mass flow rates. Furosemide, midazolam, and saline were simultaneously infused. A filter was added at the end of the infusion line to retain visible particles. Two infusion conditions were tested with and without visible particles. A partial least square method on UV spectra was used to determine simultaneously the concentrations of the two drugs at the egress of the terminal extension line. The drug mass flow rate (expressed as mg/h) was calculated as the product of drug concentration versus total flow rate. Observed/theoretical mass flow rate ratios for each drug (%) were determined per infusion condition.

**Results:**

Even in the absence of visible particles, precipitation of furosemide led to a drug loss estimated at between 10% and 15%. Furosemide is more impacted by interaction because the pH of the mixture is acid and this form is poorly soluble in an aqueous solution.

**Conclusions:**

Physical incompatibility between furosemide and midazolam leads to a significant reduction in drug delivered to the patient and may result in treatment failure.

## Background

Incompatibilities between drug solutions can jeopardize the safety and effectiveness of intravenous drug therapies, especially in the field of anesthesia and intensive care therapy [1-3]. In fact, patients in intensive care units receive many drugs simultaneously but through limited venous accesses. Several intravenous therapies have to be administered through the same catheter, thus increasing the risk of physicochemical incompatibility.

Physical incompatibilities result in visible (precipitate, color change, gas production) and invisible (subvisible particles, variations in pH) reactions. Chemical incompatibilities can lead to a decrease in drug delivery, drug degradation, and/or production of toxic products. Physicochemical incompatibilities have been reported in a series of observational studies in intensive care units [4-7]. The use of separate venous access sites can prevent contact between incompatible drugs, but all too often there are fewer venous accesses than the number of drugs infused. Filters also can be placed on the infusion line to prevent drug particles from being administered to the patient. However, they will not be able to prevent a drop in drug mass flow rates resulting from incompatibility.

The purpose of this work was to assess and to quantify the impact of physical incompatibility on the mass flow rates of drugs infused simultaneously to the patient using a single-lumen catheter, through an in vitro study.

## Methods

We have chosen to study furosemide-midazolam incompatibility, because these drugs are widely used in anesthesia and intensive care units. This incompatibility is due to an acid–base reaction. In an equivolume (1:1) mixture, the formation of a visible milky-white precipitate is immediate [8]. Because furosemide-midazolam incompatibility is pH-dependent, the impact of furosemide concentration is predictable. Furosemide in 10 mg/mL of saline is an alkaline solution (pH = 8.77). Mixing a furosemide solution with an acidic solution (i.e., 5 mg/mL midazolam, pH = 3.47) decreases the pH of the mixture sufficiently to cause furosemide precipitation [9]. Furosemide and midazolam concentrations were simultaneously determined in the solution by UV spectrophotometry coupled with partial least square (PLS) regression [10].

**Figure 1:**
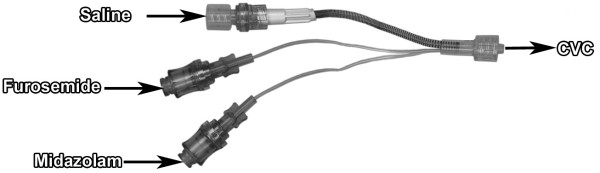
Three-lumen infusion device (VSET + M) with the central lumen reserved for carrier fluid and the two flexible low dead volume tubes reserved for furosemide and midazolam infusions.

Furosemide (10 mg/mL Furosemide, Renaudin, France), midazolam (5 mg/mL Midazolam, Mylan, France), and saline (500 mL Freeflex®, Fresenius Kabi, France) were simultaneously infused using syringe pumps connected to a three-lumen infusion device (VSET + M, Doran International, France) consisting of a central tube with an antireflux valve for saline and two flexible low dead volume tubes reserved for the furosemide and midazolam infusions (Figure 1). An extension line (diameter = 1 mm, length = 25 cm) simulating the central venous catheter was added at the distal end of the infusion set. A 1.2-μm porosity filter (Lipipor TNA, Pall, France) was added or not at the end of the infusion line. Three 50-mL syringes were prepared for each experiment: one filled with furosemide diluted in saline at 10 or 2.5 mg/mL, one filled with midazolam diluted in saline at 1 mg/mL, and one with saline only. The final drug concentration was checked using a spectrophotometric method before infusion.

Using unpublished results, we defined two infusion conditions leading or not to visible particle formation. The choice of drug concentrations was in accordance with clinical practice and infusion sets were used with no filter:

1) 10 mg/mL of furosemide at 2 mL/h infusion rate, 1 mg/mL of midazolam at 2 mL/h, and saline at 100 mL/h (condition leading to visible particle formation).

2) 2.5 mg/mL of furosemide at 8 mL/h infusion rate, 1 mg/mL of midazolam at 2 mL/h, and saline at 50 mL/h (condition not leading to visible particle formation).

The infusion was first subjected to visual inspection, and then, in the absence of visible particles, a 25-mL sample was collected at the egress of the extension line. Particle counts were taken using a particle counter (APSS-2000, PMT, France). Tests were performed under the conditions described in Chapter 2.9.19 of the 7.5^th^ European Pharmacopeia [11]. The infusion condition complied with the subvisible particle count test for a high volume as long as the average number of particles present in the sample tested did not exceed 25 per mL for particle sizes ≥10 μm and 3 per mL for particle sizes ≥25 μm. Each infusion condition was subjected to the visual inspection test three times. Three particle counts per sample were performed for the condition not leading to visible particle formation. All tests were performed at room temperature between 18°C and 22°C.

Our study was divided into two parts. In the first, we revalidated the two infusion conditions, following the same methods. In the second, for each infusion condition tested, we determined the mass flow rates of furosemide and midazolam on the infusion line with and without filter. Five trials were made per infusion condition tested. Drug concentrations in the mixture at the egress of the infusion line were determined using UV spectrophotometry (model UV-2450, Shimadzu, France) and partial least square (PLS) analysis. All information from the spectrophotometer was collected with UVProbe 2.21 software (Shimadzu, France). A partial least square (PLS) method on UV spectra was used to determine simultaneously the concentrations of the two drugs at the egress of the terminal extension line.

PLS regression is a simple and powerful multivariate method based on factor analysis and is used for building regression models based on latent variable decomposition relating a block of independent variables, x (spectra), to a block of dependent ones, y (concentrations). PLS regression was obtained using the PLS module of XLSTAT software version 2011.2.01 (Addinsoft, France). The 220–320 nm spectral zone was used to obtain the best model. The recovery percentage was in the 100.14–101.25% range. Detection limits (LOD = 3.3 x standard deviation/gradient) of drugs in mixtures were 0.19 μg/mL for furosemide and 0.36 μg/mL for midazolam. The quantification limits (LOQ = 10 x standard deviation/gradient) were 0.57 μg/mL for furosemide and 1.10 μg/mL for midazolam. Selectivity, calculated from the net analyte signal, was equal to 0.18 and 0.2 for furosemide and midazolam respectively. Because these exceeded the spectrophotometer’s linear range, they were diluted in saline. The drug mass flow rate (expressed as mg/h) was calculated as the product of drug concentration against total flow rate. Observed/theoretical mass flow rate ratios for each drug (%) also were determined per infusion condition. Measurements of pH were performed on drug solutions at the egress of infusion device using a pH meter (PHM201 MeterLab, Radiometer Analytical, Villeurbanne, France).

The Student’s *t* test was used to compare observed and theoretical drug mass flow rates and filtered and nonfiltered data after performing the Shapiro-Wilk test to check that the data observed was normally distributed. Results are expressed as mean values ± standard deviations (± SD) of mass flow rates. The level of significance was established at 0.05.

## Results and Discussion

The results are presented in Table 1. For the infusion condition that did not lead to the formation of visible particles, the number of subvisible particles was in accordance with the threshold defined by the European Pharmacopeia (18.6 and 0.4/mL for particles ≥ 10 μm and ≥ 25 μm, respectively).

**Table 1:** Determination of drug mass flow rates for the two infusion conditions tested

	**Infusion condition not leading to visible particle formation**		**Infusion condition leading to visible particle formation**	
**Furosemide**				
Concentration in syringe (mg/mL)	2.5		10	
Flow rate (mL/h)	8		2	
Theoretical mass flow rate (mg/h)	20		20	
**Midazolam**				
Concentration in syringe (mg/mL)	1		1	
Flow rate (mL/h)	2		2	
Theoretical mass flow rate (mg/h)	2		2	
Saline flow rate (mL/h)	50		100	
pH value of mixed solution at the egress of the infusion device	5.60 (±0.11)		5.62 (±0.07)	
**Particles**				
Visible	No		Yes	
Subvisible (in accordance with the EP threshold)	Inferior		Not applicable	
		
Average number of particles ≥10 μm	18.6			
Average number of particle ≥25 μm	0.4			
**Furosemide**	**With filter**	**Without filter**	**With filter**	**Without filter**
Observed mass flow rate (mg/h)	18.1 (±0.77)*	18.64 (±0.28)*	17.18 (±0.7)**	17.12 (±0.63)***
Ratio Observed/Theoretical (%)	90 (±4)	93 (±1)	86 (±4)	86 (±3)
**Midazolam**	**With filter**	**Without filter**	**With filter**	**Without filter**
Observed mass flow rate (mg/h)	1.91 (±0.26)	1.92 (±0.24)	2.08 (±0.26)	1.98 (±0.3)
Ratio Observed/Theoretical (%)	95 (±13)	96 (±12)	104 (±13)	99 (±15)

*P = 0.005 (observed vs. theoretical value).

**P = 0.001 (observed vs. theoretical value).

***P < 0.001 (observed vs. theoretical value).

Results are expressed as mean values ± standard deviations (SD) of mass flow rates and pH values. Observed/theoretical mass flow rate (%) ratios were also determined for each drug per infusion condition.

Furthermore, for both drugs, the observed mass flow rates varied little with or without filter on the infusion set. For midazolam, observed mass flow rates were similar to theoretical values (ratios close to 100%) whatever the infusion conditions. For furosemide, however, observed mass flow rates were significantly different from theoretical values whatever the infusion conditions. The lowest furosemide mass flow rate value was found for the infusion condition leading to visible particles where a 14% loss was observed compared with theoretical values (ratios equal to 86%). Even in the absence of visible particles, a furosemide loss of approximately 7–10% compared with theoretical values was noted (ratios equal to 90% and 93% with filter and without filter, respectively). For both infusion conditions, the addition of a filter to the infusion set had no effect on the observed mass flow rates of the two drugs.

This study highlights the reduction in drug mass flow rates when physical incompatibility occurs between two drugs. Preventing incompatibility is important if injectable drugs are to be safely administered. In “high-risk” cases where drug incompatibility is very probable, the use of a filter protects the patient against particle emboli. Nevertheless, the filter does not prevent drug loss in the case of precipitation. In this study, furosemide precipitation resulting in the formation of visible and/or subvisible particles led to a drug loss to the patient estimated at between 10% and 15%. This was not caused by interaction with the filter, because similar results were obtained with or without filter. Furosemide was more impacted by the interaction of the two drugs as the pH of the mixture is acid (Table 1), and furosemide in its acidic form is poorly soluble in an aqueous solution [12]. For a substance to be soluble in water, it must be in its ionized form, which is not the case here for furosemide [9]. The impact of mixing conditions (concentration and flow rates) on particle formation also must be taken into consideration as the pH levels of mixed solutions were similar at the end of the extension line. This reduction in drug delivery should not have any clinical impact in the case of furosemide. Nevertheless, this result raises the issue for other mixtures of acid (amiodarone, ciprofloxacin, dobutamine, midazolam, norepinephrine) and alkaline (acyclovir, furosemide, phenytoin) drug solutions that may lead to physical incompatibilities. The major consequence for the patient could be therapeutic failure, especially in the case of drugs with a narrow therapeutic index.

It should be noted that there are several limitations to this study. Our assessment was limited to a two-drug combination inducing pH-dependent incompatibility. The results may vary with other drug combinations. Our results should be confirmed by testing other combinations of incompatible drugs and more than two drugs among those commonly used in intensive care and anesthesia.

## Conclusions

Physical incompatibility between two drugs may lead to a significant reduction in drug amount delivered to the patient even in the absence of precipitate. Physicians, nurses, and pharmacists must be sensitive to the perfusion conditions of simultaneously infused drugs. The absence of precipitate does not necessarily mean the absence of reaction between drugs.

## Competing interests

Pascal Odou, Bertrand Décaudin, and Bertrand Debaene report receiving reimbursements of travel expenses related to medical congresses from Doran International.

## Authors’ contributions

AF helped analyze the data, write the manuscript, and collect data. BD helped design the study, conduct the study, analyze the data, and write the manuscript. CB helped write the manuscript. BD helped design the study, analyze the data, and write the manuscript. PO helped design the study, conduct the study, analyze the data, and write the manuscript. All authors have read and approved the final manuscript.
